# Exposure to Endocrine Disruptor Induces Transgenerational Epigenetic Deregulation of MicroRNAs in Primordial Germ Cells

**DOI:** 10.1371/journal.pone.0124296

**Published:** 2015-04-21

**Authors:** Miguel A. Brieño-Enríquez, Jesús García-López, David B. Cárdenas, Sylvain Guibert, Elouan Cleroux, Lukas Děd, Juan de Dios Hourcade, Jana Pěknicová, Michael Weber, Jesús del Mazo

**Affiliations:** 1 Department of Cellular and Molecular Biology, Centro de Investigaciones Biológicas (CSIC), Madrid, Spain; 2 Biotechnology and Cell Signaling, CNRS UMR7242, University of Strasbourg, Strasbourg, France; 3 Institute of Biotechnology AS CR, v. v. i., Prague, Czech Republic; Seoul National University, KOREA, REPUBLIC OF

## Abstract

In mammals, germ cell differentiation is initiated in the Primordial Germ Cells (PGCs) during fetal development. Prenatal exposure to environmental toxicants such as endocrine disruptors may alter PGC differentiation, development of the male germline and induce transgenerational epigenetic disorders. The anti-androgenic compound vinclozolin represents a paradigmatic example of molecule causing transgenerational effects on germ cells. We performed prenatal exposure to vinclozolin in mice and analyzed the phenotypic and molecular changes in three successive generations. A reduction in the number of embryonic PGCs and increased rate of apoptotic cells along with decrease of fertility rate in adult males were observed in F1 to F3 generations. Blimp1 is a crucial regulator of PGC differentiation. We show that prenatal exposure to vinclozolin deregulates specific microRNAs in PGCs, such as *miR-23b* and *miR-21*, inducing disequilibrium in the *Lin28*/*let-7*/*Blimp1* pathway in three successive generations of males. As determined by global maps of cytosine methylation, we found no evidence for prominent changes in DNA methylation in PGCs or mature sperm. Our data suggest that embryonic exposure to environmental endocrine disruptors induces transgenerational epigenetic deregulation of expression of microRNAs affecting key regulatory pathways of germ cells differentiation.

## Introduction

Primordial germ cells (PGCs) are the embryonic precursors of the germ cell lineage [1]. PGC specification depends on the key factors BLIMP1 (PRDM1) and PRDM14 that induce repression of the somatic program, epigenetic reprogramming and re-expression of pluripotency genes. Development of PGCs also requires the RNA-binding factor LIN28 that binds to specific microRNA (miRNA) precursor: the *let-7* pri-miRNA preventing the processing into mature forms of *let-7* miRNAs. In absence of LIN28, *let-7* miRNAs are overexpressed in PGCs and bind to the 3′UTR of the *Blimp1* mRNA, which blocks its translation and inhibits PGC development [2].

In mouse, PGC precursors are specified in the epiblast around 6.25 days post coitum (dpc) [3]. Thereafter, PGCs proliferate and migrate through the hindgut endoderm to enter the genital ridges at day 10.5 and colonize the fetal gonads where they continue to proliferate until day 13.5 [4]. During this period, PGCs undergo global epigenetic reprograming characterized by the erasure of DNA methylation and histone modifications [5]. After the onset of gonadal sex determination, the PGC genome initiates re-methylation of DNA accompanied by remodeling of histone modifications in a sex specific manner [5,6]. Genetic and epigenetic changes during reprogramming of embryonic germ cell precursors make the prenatal period a sensitive window for potential adverse effects caused by environmental factors. The environmentally induced changes produced at this period are capable of inducing adult onset diseases than can also be perpetuated across multiple generations by transmission through the germ line (transgenerational epigenetic inheritance) [7]. Epigenetic mechanisms, including DNA methylation, histone modifications and specific miRNAs expression have been proposed to mediate such transgenerational transmission [8,9].

Endocrine disruptors (EDs) are synthetic or natural substances that alter the homeostasis of the endocrine system. Vinclozolin (VCZ) (3-(3, 5-dichlorophenyl)-5-methyl-5-vinyl-oxazolidine-2, 4-dione) is a widely used fungicide with antiandrogenic effects in mammals. VCZ metabolites are competitive antagonists of androgen receptor (AR) ligand binding [10]. Several studies performed in rodents (mainly rats) showed that exposure to VCZ induces masculinized females, feminized males [11], decreased sperm number and increased apoptosis in the seminiferous tubule cells [12], and abnormal fertility rates [13]. Some of the effects of VCZ have been observed to be passed to subsequent unexposed generations, which are hypothesized to be caused by the gametic transmission of deregulated epigenetic marks such as altered DNA methylation [12,14–16]. Environmental exposure to chemicals can induce aberrant microRNA (miRNA) expression [17]. miRNAs are small non-coding RNAs (~21–23 nt) acting as potent post-transcriptional regulators of target mRNAs [18]. Some studies have established that miRNAs can interplay with epigenetic regulators and can also be epigenetically regulated [19,20].

In the present study, we analyzed the effects of prenatal exposure to VCZ in mice. We evaluated the effects of VCZ in the first generation of exposed animals as well as the transgenerational transmission through the male germline in subsequent non-exposed generations (F1 to F3). We describe that prenatal exposure to VCZ induces a perturbation of apoptosis and fertility that persist over three generations in male mice. We provide evidence that this transgenerational phenotype is not associated with major changes in gametic DNA methylation but is associated with long lasting deregulations of several miRNAs in male PGCs, in particular the *Lin28*/*let-7*/*Blimp1* pathway that plays important roles in PGC specification and development.

## Results

### Transgenerational phenotypic consequences of prenatal exposure to vinclozolin in males

Oral intake is the most common way of VCZ exposure in human. To mimic this mechanism of exposure in mouse, pregnant females were exposed to VCZ by oral intake (in the drinking water) during the entire duration of pregnancy with two different doses: a low dose (1mg/kg bw/d) (VD1) and a high dose (100mg/kg bw/d) (VD2). The offspring obtained from these exposed females were denominated F1 animals. F2 animals were obtained from the mating of F1 males with unexposed females, and F3 animals from the mating of F2 males with unexposed females (S1 Fig).

**Fig 1:**
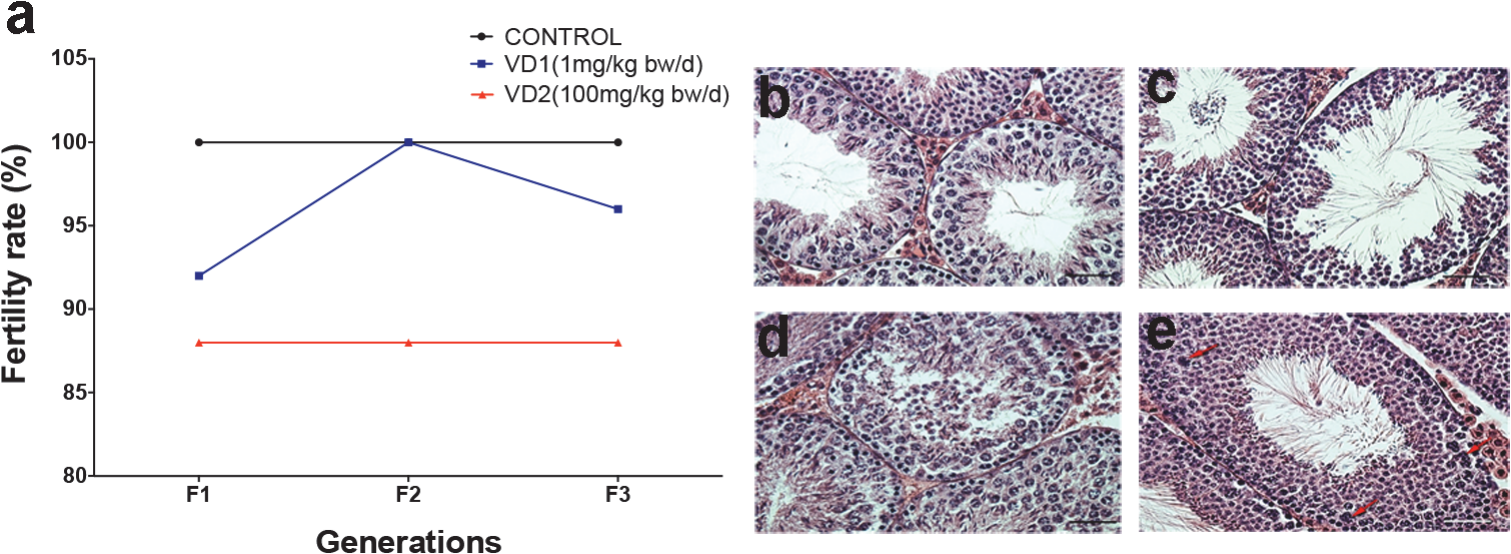
Fertility and histopathological analysis in the testis of mice exposed to VCZ. a) Fertility rate after fetal exposure to the low dose (VD1) or the high dose (VD2) of VCZ, expressed as a percentage of fertile males along the three generations (F1, F2 and F3). b-e) Histological analysis of testis sections stained by hematoxilyn-eosin from 10 weeks old mice from the control group (b) or VCZ exposed group (c-e), show examples of impairment of seminiferous epithelium tubule (c), tubule disintegration with cells in the lumen (d), and hypertrophic cells with fragmented karyoplasm (e, red arrows).

We first performed a phenotypic analysis of adult male mice in all three generations (F1 to F3). We found no differences in body weight, testis weight, and other morphometric markers between VCZ exposed and control animals (data not shown). However, we observed that the male fertility rate is reduced by 8% in VD1 and 12% in VD2 F1 compared to the control group (Fig 1A). The fertility rates of animals of VD1 group recovered to values comparable to control animals along the next generations (F2 and F3). In contrast, the males from high dosage group (VD2) showed reduction of 12% in their fertility index in all the generations analyzed (F1-F3) (Fig 1A).

**Fig 2:**
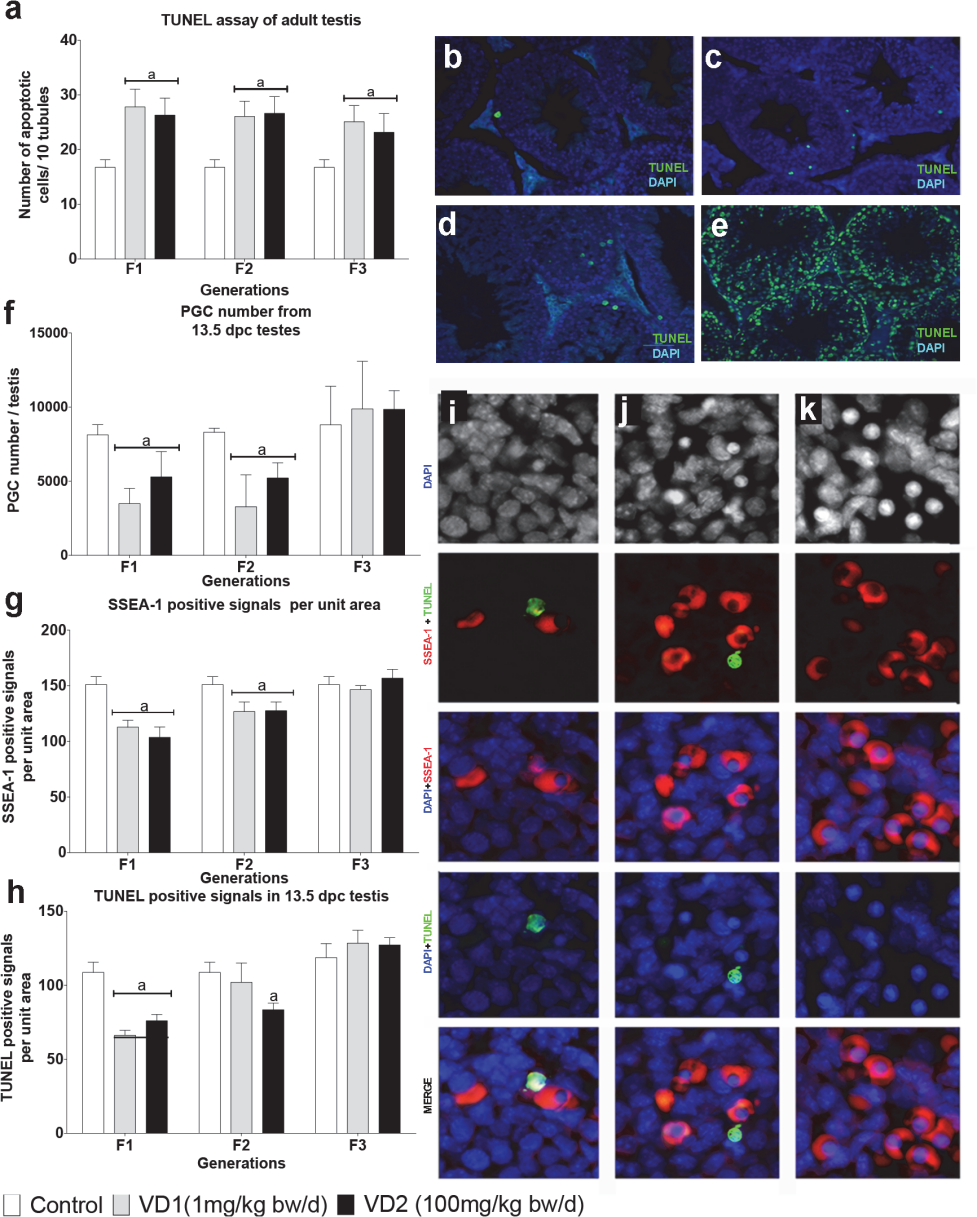
Apoptosis in adult testis and PGCs of mice exposed to VCZ. a) Number of apoptotic cells counted per 10 tubules in the adult testis of control, VD1 and VD2 in F1 to F3 generations. b-d) Examples of apoptosis by TUNEL in testis sections of control (b), VD1 (c) and VD2 (d) animals. e) TUNEL positive signals after DNAse treatment of testis sections is shown as a positive control. f) Total number of PGCs isolated per testis by cell sorting with the surface marker SSEA-1 from control, VD1 and VD2 13.5dpc embryos. g) Histological evaluation of the number of PGCs in testis of 13.5dpc embryo by immunostaining with the marker SSEA-1. h) Evaluation of apoptosis in 13.5 dpc testis by TUNEL assay. i-k) Examples of co-detection of apoptosis by TUNEL and SSEA-1 positive PGCs cells by confocal microscopy analysis. i) Apoptosis detected in a PGC. j) Apoptosis detected in a somatic cell. k) None apoptotic cell detected. In the histograms, (a) indicates a significant statistical difference compared to the control value (p<0.01), and the error bars represent the standard deviation (SD).

To evaluate whether the reduction in the fertility index was associated to tissue changes in the testis, we monitored histopathological anomalies and apoptosis in the seminiferous epithelium from adult testis from F1 to F3. Histological analysis showed an increased number of impairment of seminiferous epithelium tubule and hypertrophic cells with fragmented karyoplasm in the lumen of tubules in the three generations of exposed males (Fig 1B–1E). The evaluation of apoptotic cells by the TUNEL method revealed a significant increase of apoptosis in seminiferous tubules of adult testis in all the generations and with both doses (Fig 2A and 2B–2E) (p≤0.0001). Surprisingly, both groups VD1 and VD2 showed similar increments in apoptosis (1,38 to 1,50 fold compared to control animals).

To determine if the gonadal defects in exposed animals are a consequence of dysfunctions that arise in embryonic germ cells, we analyzed PGCs isolated at 13.5 dpc using the surface marker SSEA-1. The results revealed that VCZ exposure induces a decrease in the number of PGCs recovered per testis. Compared to controls, F1 animals from the VD1 and VD2 groups showed reduction of 57% and 35% respectively in the number of PGCs (p≤0.001) (Fig 2F). The reduction in the PGC number was similar in F2 males with a reduction of 61% in VD1 and 38% and VD2 (p≤0.001) (Fig 2F). However, the number of PGCs recovered normal levels in VD1 and VD2 groups in the F3 generation (Fig 2F). To corroborate these findings, we performed immunofluorescence against SSEA-1 in sections of 13.5dpc testis. The number of positive SSEA-1 signals per unit area is significantly lower in F1 and F2 13.5dpc testis from exposed animals compared to controls (Fig 2G). In summary, both experimental approaches indicated a significant reduction in the number of PGCs at 13.5dpc in F1 and F2 males exposed to VCZ.

To uncover the potential differences in the PGCs apoptotic rate among control and experimental samples, co-localization analysis of the TUNEL and SSEA-1 positive cells were performed using NIS elements picture analyzer (Fig 2I–2K). The signals from corresponding TUNEL and SSEA-1 fluorescence channels in individual testis embryo sections were measured and the correlation of the fluorescent signal intensity was computed. The results were outputted as Mander's overlap (MO) coefficient and Pearson's correlation coefficient (r) [21]. The results showed that there was a significantly higher overlap (MO) of the TUNEL and SSEA-1 fluorescent signals in three generation exposed to high doses of VCZ. The results showed a decrease of positive signals in F1-VD1 and VD2 as well as in F2VD2 (Fig 2H). The mean value of Rp- Pearson’s correlation (Rp) in control was 0.72; representing that 51% of the apoptotic signals (TUNEL) were coincident with SSEA-1 signal. Testis from VCZ exposed mice showed a significantly increment, reaching the maximum in F1VD2 (Rp = 0. 90) representing that 80% of co-localized signal. These results indicate that PGCs from groups exposed to VCZ have significantly higher probability to undergo apoptotic process compared to the PGCs from non-exposed animals. The differences in positive TUNEL cells detected in F3 between adult testis and PGCs suggested that in addition to germ cells somatic cells of testis could also be affected by VCZ exposure.

### Prenatal exposure to vinclozolin induces a transgenerational perturbation of miRNAs involved in PGC differentiation

**Fig 3:**
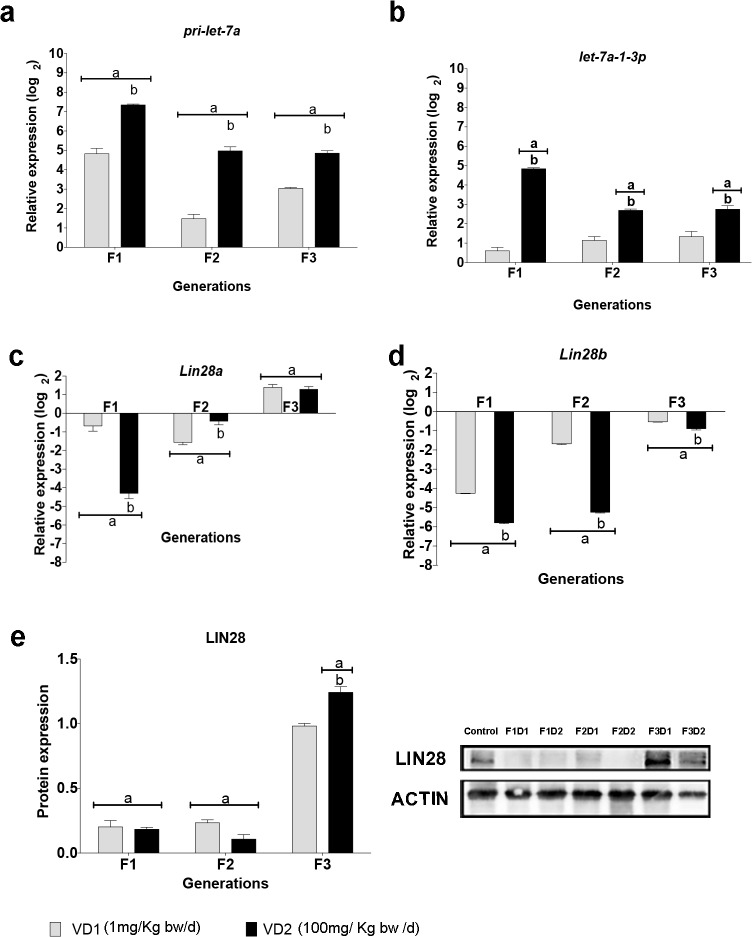
Expression of *pri-let-7a*, *let-7a-1-3p* and *Lin28* in PGCs of mice exposed to VCZ. a-d) The graphs show the log_2_ of the fold change of expression in PGCs of exposed embryos relative to the unexposed control embryos. e) LIN28 protein levels measured by Western blot in 13.5 dpc PGCs. The graph bars show the quantification of protein levels normalized to the unexposed control (value = 1). In the graphs the error bars represent the standard deviation (SD), (a) indicates a significant statistical difference of VD1 and VD2 compared to the control (p≤0.01), (b) indicates a significant statistical difference of VD1 compared to VD2 (p≤0.01).

**Fig 4:**
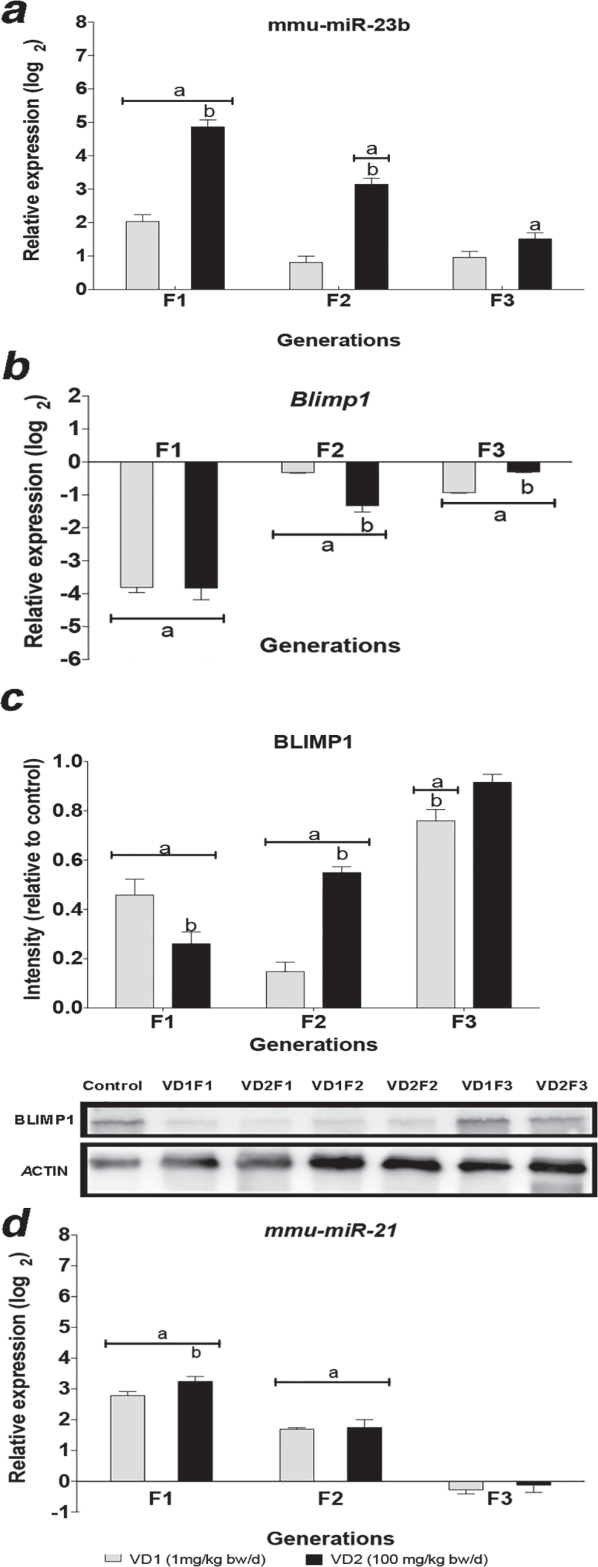
Levels of expression of *Blimp1* and regulatory miRNAs in PGCs of mice exposed to VCZ. a) Relative expression of *miR-23b* in PGCs of exposed embryos relative to the control embryos. b-c) Relative expression of the *Blimp1* mRNA (b) and BLIMP1 protein (c) in PGCs of exposed embryos relative to the control embryos. d) Relative expression of *miR-21*. The error bars represent the standard deviation (SD), (a) indicates a significant statistical difference of VD1 and VD2 compared to the control (p≤0.01), (b) indicates a significant statistical difference of VD1 compared to VD2 (p≤0.01).

Using miRNA TaqMan-Megaplex arrays (Applied Biosystems), we profiled the differential expression of miRNAs in PGCs from VCZ-exposed and control F1 mice (data not shown). We then selected candidate miRNAs owing to their reported or predicted role in germ cell development and differentiation. This includes members of the *let-7* family and *miR-23b*, which can regulate *Blimp1*, a key in the PGC differentiation [22–24]. *let-7* and *mir-23b* also regulate *Lin28*, which encode a stem cell-expressed RNA binding protein required for PGC development. In turn, LIN28 establishes a feedback loop by binding to *let-7* pre-miRNAs and pri-miRNAs and inhibiting their processing into mature miRNAs [25–27]. In mammals, two *Lin28* paralogs are expressed (*Lin28a* and *Lin28b*), which are functionally equivalent but with different expression patterns, cellular localization and different mechanism of *let-7* repression [28]. We quantified the expression levels of *let-7* miRNA and target mRNAs in samples from all three generations. Our results showed that exposure to VCZ increased significantly the expression of the precursor and some mature forms of *let-7* in F1 to F3 (p≤0.001) (Fig 3A and 3B). The increased levels of *let-7* in PGCs correlate with a downregulation of the *Lin28* paralogs. We found that *Lin28a* is downregulated in PGCs from exposed F1 and F2 animals, whereas its expression was slightly increased in F3 in an unexpected rebound-like effect. Similarly, *Lin28b* expression levels were decreased in PGCs from F1 and F2 animals (p≤0.001), whereas the levels tend to normalize in F3 (Fig 3C and 3D). To corroborate these findings, we performed Western blot analysis of LIN28 in PGCs and observed a decrease of protein levels in F1 and F2 exposed animals and a recovery to normal levels in F3 (Fig 3E). In addition to *let-7*, *Lin28* is also a predicted target of *miR-23b*. We found that *miR-23b* was upregulated in a dosage-dependent manner in PGCs from the three generations of embryos exposed to VCZ (Fig 4A).

In addition to Lin28, *let-7* and *miR-23b* are also potential regulators of *Blimp1*. Therefore we next asked whether the changes in miRNA expression in PGCs were associated with changes in the expression of *Blimp1*. We quantified *Blimp1* mRNA by RT-qPCR and found strong downregulation of *Blimp1* transcripts (p≤0.001) in PGCs from the three generation of exposed animals (Fig 4B). In accordance with the mRNA levels, the Western blot analysis confirmed the decrease of BLIMP1 in PGCs from the three generations of exposed animals (Fig 4C). Altogether, our data show that the transgenerational upregulation of *let-7* and *miR-23b* in PGCs of VCZ exposed animals was associated with the downregulation of the key PGC factors *Lin28* and *Blimp1* along three generations.

To explore whether other miRNAs and genes involved in germ cell development were altered in PGCs as consequence of VCZ exposure, we examined the expression of *miR-21*, miR-135*, *miR-381* and *miR-486* miRNAs. We found that VCZ led to elevated *miR-21* levels in PGCs from F1 and F2 but not F3 animals (Fig 4D). *miR-21* has been implicated in the regulation of spermatogonia stem cells in mice [29] and its transcription is regulated by BLIMP1 [30]. Not other changes were observed.

Finally, terminal uridylyl transferases (TUTases) interact with LIN28 to induce poly-uridylation of *let-7* precursors at their 3’ end, which interferes with *let-7* maturation and facilitates the miRNA precursor degradation by the recruitment of exonucleases [31]. *TUTase4* (*Zcchc11*) and *TUTase7* (*Zcchc6*) also promote the mono-uridylation of *pre-let-7* in absence of LIN28, which facilitates DICER processing and increases the levels of mature *let-7* [32]. To investigate whether the deregulation of the *Lin28*/*let-7* pathway detected in PGCs was associated to the deregulation of TUTases, we performed RT-qPCR analysis of *TUTase4* and *TUTase7*. Surprisingly, both genes were found significantly overexpressed in PGCs of VCZ-exposed mice in the three generations (p≤0.001) (S2 Fig). Together, these results suggest that a coordinated deregulation of *Lin28* and TUTases participate in the upregulation of *let-7*.

**Fig 5:**
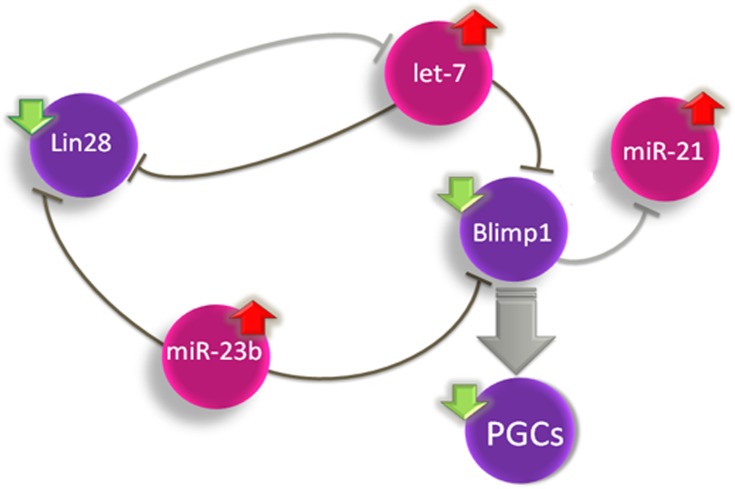
Schematic model for the deregulation of PGC development by the *Lin28*/*let-7*/*Blimp1* pathway in the PGCs from mice exposed to VCZ. Red arrows indicate upregulation and green arrows indicate downregulation. miRNAs are illustrated in pink. A consequence of the downregulation of BLIMP1 is the reduction in the number of PGCs in embryonic testis.

Altogether, these results indicate that in the mouse, the exposure to VCZ, even at low dosage, disturbs the expression of several miRNAs and the balance of the *Lin28/let-7/Blimp1* pathway during the specification of PGCs, which provides a possible molecular explanation for the perturbation caused in germ cell precursors (Fig 5). Remarkably, this disturbance is transgerationally transmitted up to the F3 generation.

### Impact of vinclozolin exposure on DNA methylation profiles in PGCs and spermatozoa from F1 males

**Fig 6:**
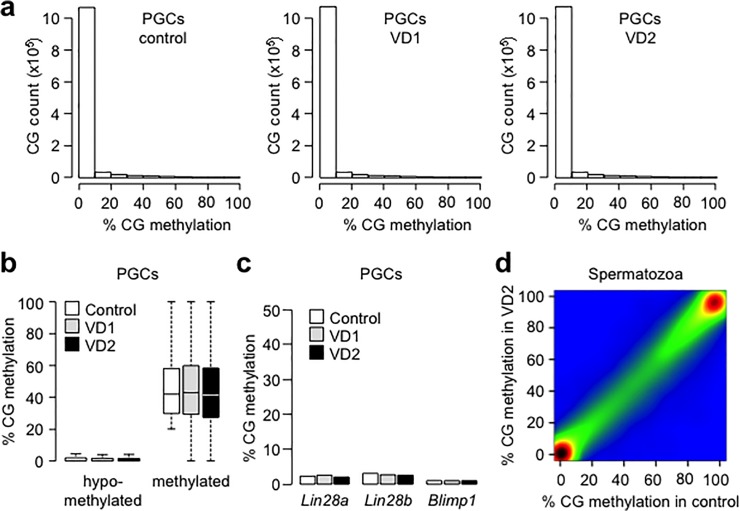
DNA Methylation analysis in PGCs and spermatozoa of F1 mice exposed to VCZ. a) Distribution of the percentages of CpG methylation measured by RRBS in 13.5 dpc PGCs from control, VD1 and VD2 exposed embryos at the F1 generation. b) Distribution of the CG methylation measured in sequences hypomethylated in normal PGCs (defined as <20% methylation in control PGCs) or partially resistant to demethylation in PGCs (defined as >20% methylation in control PGCs). c) Evaluation of the methylation status of the promoters of *Lin28a*, *Lin28b* and *Blimp1* in 13.5dpc PGCs by single locus bisulfite sequencing (for *Lin28a* and *Lin28b*) and RRBS (for *Blimp1*). d) Pairwise comparison of CpG methylation measured in 400bp tiles in adult spermatozoa isolated from F1VD2 males compared to control males, which reveals global conservation of DNA methylation (Pearson correlation coefficient r = 1). The density of points increase from blue to dark red.

Previous studies performed in rodent models proposed that the transgenerational effects caused by environmental factors such as VCZ are mediated by the transmission of altered gametic DNA methylation [12,15,16]. To determine whether vinclozolin influences patterns of gametic DNA methylation in our experimental model, we generated quantitative maps of methylation for around 1,3 million CpGs using the reduced representation bisulfite sequencing (RRBS) technique (S1 Table). We first asked whether VCZ interferes with epigenetic reprogramming in PGCs by generating RRBS maps in 13.5 dpc PGCs from control and exposed F1 males. As expected, control PGCs showed a massive erasure of DNA methylation characteristic of these cells [5]. However, this global demethylation pattern is not altered in PGCs from F1 males exposed to the low dose or the high dose of VCZ (Fig 6A). The rare sequences that retain methylation in control PGCs, which are mostly associated with transposable elements, have similar methylation in PGCs from exposed animals (Fig 6B). We investigated methylation at genes involved in germ cell function and development and found no evidence for abnormal methylation in PGCs from exposed animals, including in the promoters of genes deregulated in PGCs (*Lin28a*, *Lin28b*, *Blimp1*) (Fig 6C). Taken together these results indicate that VCZ does not interfere with the process of DNA methylation reprogramming in early PGCs in our experimental model and deregulates PGC developmental pathways independently of DNA methylation.

We next asked whether abnormal DNA methylation accumulates during later stages of spermatogenesis in male mice exposed to VCZ. We focused on F1 males exposed to the high dose of VCZ because this dose induces the strongest transgenerational phenotype. We generated RRBS methylomes in mature spermatozoa isolated from exposed F1 males and several control pools (S1 Table). We observed identical global profiles of CG methylation in control and exposed animals, indicating that VCZ has a minor impact on sperm methylation (Fig 6D). We identified only very few sequences with variable methylation between samples, most of them being already variable in control samples and therefore likely representing natural variations in the outbred CD-1 strain (data not shown). We only identified one sequence with a consistent reduced methylation in exposed compared to control samples, located in the exon 18 of the *Paok3* gene (S3A Fig). Interestingly, it was reported that *Paok3* can be regulated by androgen in human cells [33]. In contrast to results obtained with another model of mice exposed to VCZ [14], we found no evidence that VCZ modifies DNA methylation at sequences carrying long term epigenetic memory such as imprinting control regions (S3B Fig). Therefore our data indicate that the exposure to VCZ does not induce major changes in gametic DNA methylation, suggesting that mechanisms other than DNA methylation participate in the transgenerational transmission of VCZ-induced phenotypes.

## Discussion

This work demonstrates that the fetal exposure of mice to VCZ induces trans-generational changes in miRNAs and target gene expression profiles of the *Lin28/let-7/Blimp1* pathway in PGCs, even at a dosage under those considered as NOAEL “No Observed Adverse Effect Level” (established at 1.2 mg/kg/day) (http://www.epa.gov/oppsrrd1/REDs/2740red.pdf).

It has been reported that intraperitoneal exposure of female rodents to VCZ during gestation (8–15 dpc) induces spermatogenic cell apoptosis, decreased sperm count and motility, histological abnormalities and altered DNA methylation patterns along four generations [12,15,34,35]. In the present study, mice exposed to VCZ, administered orally during the entire pregnant period, showed an increased rate of apoptosis in adult testis and reduced male fertility in three generations. In contrast, we did not observe differences in weight, sperm number or other histological patterns in adult offspring mice. We also detected a significant increase in the apoptosis of PGCs that could be caused by imbalanced signals between the effects of androgens in fetal germ cells [36] and the antiandrogenic action of VCZ, which could determine the dysfunctional phenotypes observed in adulthood. Differences observed in our study compared to previous reports could also be related to the method and time of exposure. Oral intake mimics the human exposure to VCZ entails different mechanism of absorption, distribution and degradation of the compound from intraperitoneal injection. Interestingly, the phenotypic consequences of exposure are observed with both doses of VCZ and in most cases do not show a dose-response effect. These results provide further support to the theory that the exposure to environmental toxicants during fetal development can induce dysfunctions and diseases in adulthood and even in the next generations, the so-called fetal origin of adult diseases [37].

MicroRNAs modulate gene expression and play key roles in developmental processes, regulation of cell self-renewal, cellular differentiation, proliferation, and apoptosis. However, the functional importance of miRNAs in the reprotoxicity caused by VCZ has not been investigated. Our results show that VCZ disrupts the normal pattern of expression of specific miRNAs and some of their putative target genes in PGCs. Deregulated miRNAs (*let-7*, *miR-21*, *miR-23b)* and their targets genes (*Lin28a*/*Lin28b* and *Blimp1*) are involved in PGC specification and development. BLIMP1 is responsible for the specification of PGCs in the posterior epiblast and is regulated by the expression of *let-7*, which is in turn regulated by LIN28 [23,38]. Therefore, the balance among *Blimp1*, *Lin28* and *let-7* needs to be tightly controlled for the proper specification of PGCs in the mouse embryo. Our results demonstrate for the first time that VCZ induces a deregulation of the *Lin28/let-7/Blimp1* pathway in embryos. We observed a downregulation of the abundance of *Lin28* transcripts and consequently also the LIN28 protein in PGCs. As was described in *Lin28* knockdown mice [39], the reduction of LIN28 by VCZ is associated with an accumulation of precursor and mature forms of *let-7* in germ cells. At the same time, VCZ also induces the overexpression of *TUTase4* (*Zcchc11*) and *TUTase7* (*Zcchc6*) that, in absence of LIN28, facilitate the processing of *pre-let-7* into *let-7* [32] explaining the accumulation of mature *let-7* in exposed animals. Interestingly, previous studies showed that *let-7* is overexpressed after exposure to other EDs. For example, Bisphenol A (BPA) induces the overexpression of three members of *let-7* miRNA family in human placental cells [40]. As mentioned before, *let-7* family members and *Lin28* are critical regulators of cell proliferation and are considered as tumor suppressors [25,41,42]. In this sense, the upregulation of *let-7* miRNAs could be a cellular response to protect from the damage induced by environmental factors.

In accordance with the model of *Blimp1* regulation, we show that the downregulation of *Lin28* and upregulation of *let-7* are associated with a downregulation of *Blimp1* in PGCs from exposed animals. The changes observed in PGCs of mice exposed to VCZ mimic the changes observed in *Blimp1* heterozygous mutants where the partial reduction of *Blimp1* expression leads to a smaller number of PGCs in embryos which fail to show the characteristic migration and proliferation of PGCs [22,43]. This leads us to propose that the deregulation of the *Lin28/let-7* pathway and partial reduction of *Blimp1* is one molecular pathway that directly contributes to inhibit PGC development in embryos exposed to VCZ (Fig 5).

Besides the *Lin28/let-7/Blimp1*, other pathways certainly also contribute to the defects in PGC development and apoptosis caused by the exposure to VCZ. Our results show that VCZ induces an upregulation of *miR-21* and *miR-23b* in PGCs. *miR-23b* is a potential regulator of *Lin28* and *Blimp1*, an is also involved in the control of cell migration and adhesion [44], growth arrest [45] and apoptosis [46]. On the other hand, *miR-21b* is important for the self-renewal of mouse spermatogonial stem cells [29]. Studies performed in human breast cancer showed that cells to antiandrogenic chemicals overexpress *miR-21*, reducing cell proliferation and motility [47]. This suggests that *miR-23b* and *miR-21* could participate in the response to VCZ by modulating cell proliferation and migration in germ cells. The consequences of VCZ exposure on germ cell and gonadal functions are not only observed in F1 animals exposed *in utero* but are transmitted through the male germline to subsequent unexposed generations in F2 and F3. We note however that some of the phenotypic traits and gene markers tend to revert to a normal state in F3.

What could be the mechanisms of epigenetic transmission through generations? Previous studies have proposed that the epigenetic inheritance of phenotypes is mediated by the transmission of abnormal gametic DNA methylation [7,12,15,16]. In the mouse, PGCs undergo a global wave of epigenetic reprogramming during development that restores their pluripotency, which includes the erasure of somatic DNA methylation and histone posttranslational modifications [5,48]. In the male germline, PGCs then re-establish DNA methylation *de novo* and acquire the methylation profiles characteristic of mature spermatozoa between 13.5 and 17.5dpc. In the present study, we show that PGCs from F1 embryos exposed to VCZ have normal patterns of DNA methylation, which indicates that VCZ, at the dosages used, does not interfere with the global process of DNA methylation reprogramming in PGCs. Recent reports identified a small number of sequences that escape demethylation in PGCs, which are mostly associated with transposable elements [5,49]. These sequences represent prime candidates for epigenetic transgenerational inheritance, however we found that their methylation is not altered in PGCs. We also searched for patterns of abnormal DNA methylation in mature spermatozoa and found a very strong conservation of cytosine methylation marks in spermatozoa of VCZ-exposed males. In agreement with studies in mouse models of intergenerational developmental programming, we found that the reprogramming of methylation imprints in sperm is not altered by VCZ [50,51]. The apparent discrepancy with previous reports of abnormal gametic methylation after VCZ exposure may be due to strain differences, the window and dose of exposition, or the methods for methylation profiling. Several explanations can be proposed to explain the low incidence of DNA methylation anomalies. First, it is possible that changes in DNA methylation occur outside of the genomic regions covered by the RRBS method; indeed a recent survey of sperm methylation in a model of intergenerational metabolic disorder uncovered discrete sites of hypomethylation in regulatory regions distant from promoters [52], which are mostly not accessible by RRBS. Second, we cannot rule out the existence of changes in DNA methylation of small amplitude that were missed by our analysis.

Alternatively, other epigenetic marks such as histones modifications could mediate the trans-generational effects observed in VCZ-exposed mice [53]. Recent reports have shown that the mouse spermatozoa retain histones carrying specific marks of histones. This suggests that sperm modified nucleosomes, in particular those methylated on the lysine 27 of histone H3 (H3K27me3), could mediate paternal epigenetic inheritance and influence gene expression in the embryo [53]. This model is supported by a recent bioinformatics modeling approach that predicts that histone modifying enzymes play an important role in the response to endocrine disruptors such as vinclozolin and dibutyl-phthalate [54]. Finally, another possibility is the epigenetic transmission mediated by sperm miRNAs. It has been described that miRNAs can induce hereditary epigenetic variations in mice and act as the transgenerational signalling molecule called “paramutation”. Two examples of this mechanism include the epigenetic modulation of the *Kit* gene and *Sox9* gene in mice [55,56]. More recently, new studies implicated sperm small RNAs in the transgenerational transmission of paternal stress in mice [57,58]. Given that we find a number of miRNAs deregulated in germ cells as a consequence of exposure to VCZ, the possible role of miRNAs in the paternal transgenerational transmission of ED-induced phenotypes is a plausible mechanism of action that needs to be investigated in the future.

## Material and Methods

### Animals

This study was carried out in strict accordance with the recommendations in the Spanish Royal Legislative Decree RD53/2013 for the Care and Use of Laboratory Animals. The protocol was approved by the Committee on the Bioethics of Animal Experiments of the Centro de Investigaciones Biologicas (CSIC) (Permit: 23-11-11). All procedures for handling animals were in accordance with the regulations of the European Commission (Directive 2010/63/UE and Directive 86/609/ECC) and all efforts were made to minimize suffering and reducing the number of animals. CD-1 mice were supplied by our own animal facility, the CIB-CSIC bioterium. The mice were bred under pathogen-free (SPF), controlled temperature (22±1°C) and regulated humidity (50–55%) conditions with periods of light/dark of 12 h and food available ad libitum. CD-1 mice were exposed to vinclozolin by oral intake following the regimen. Mice were sacrificed by cervical dislocation. Four groups were established: control group (unexposed), vehicle group (exposed to DMSO), vinclozolin low dose group (VD1, 1 mg/kg bw/d) and vinclozolin high dose group (VD2, 100mg/kg bw/d) (S1 Fig). These estimated intakes were calculated on the basis of average drinking and body weight recorded in previous experiments performed in our lab and in agreement with the data in the literature.

The exposure to VCZ began the day of the vaginal plug, which is designated 0.5 dpc. The pregnant female mice treated with VCZ were designated as the F0 generation. The offspring of the F0 generation were the F1 generation. 20 adult females were used in each group to produce the F1 generation. At 13.5dpc 10 mothers from each group were sacrificed to recover gonads from F1 fetuses for the isolation of PGCs and the histological analysis. The remaining 10 mothers were led to give birth. After eight weeks, 25 males of the F1 generation were randomly selected and bred with 50 non-exposed females (two females per male) to obtain the F2 generation. The rest of the F1 males were sacrificed to collect samples for molecular and histopathological analysis. The same process was repeated to obtain the F3 generation. To evaluate male fertility, we randomly select 25 litters of each generation. Litters were mated with two CD1 females during one week. Female mice were checked for vaginal plugs each morning and recorded. Positive fertility was only considered after delivery.

### PGC isolation

PGCs were purified using paramagnetic technology from 13.5 dpc male embryo testes following a published protocol [59] with some modifications. Briefly, gonads from 13.5dpc embryos were dissected in EmbryoMax M2 Medium (Merck-Millipore), and testis were recognized from ovaries by their morphological appearance. Testis were separated from the mesonephros, disaggregated in 1000 μl of 0.25% trypsin-EDTA / 20 μg/ml DNase (Sigma-Aldrich) and incubated for 20 min at 37°C. The enzymatic reaction was stopped by adding M2 medium with 10% FCS (Gibco). The cells were centrifuged at 5000 rpm for 2 min and washed twice with 500 μl of M2 medium containing 10% FCS and 20 μg/ml DNase. The cell pellet was resuspended in 400 μl of M2 medium, mixed with 30 μl anti-SSEA-1 (CD15) MicroBeads (Miltenyi Biotech) and incubated for 45 min at 4°C. PGCs were isolated from stromal cells with a MS column (Miltenyi Biotech) following the manufacturer’s instructions. The purity of PGCs was verified by counting cells that stained positive for alkaline phosphatase with the naphtol AS-MX/ FAST-RED (Sigma-Aldrich) following the manufacturer’s instructions. In all cases the purity of the PGCs was 93–96%.

### Histological analysis and adult testicular tissue morphometry

From each adult mouse, one testis was fixed in 4% formaldehyde in PBS. After fixation, we prepared paraffin-embedded tissue sections (5μm thick) and stained by hematoxylin-eosine. Tissue sections were evaluated under a light microscope. In all cases, 100 seminiferous tubules were analysed by computer-assisted morphometry. The thickness of the germinal epithelium and diameter of the seminiferous tubules were measured.

### TUNEL analysis

We detected apoptotic cells in tissue sections by terminal deoxynucleotidyl transferase-mediated dUTP nick end labelling (TUNEL), using an *in situ* detection kit (Promega) according to the manufacturer’s instructions. In brief, paraffin-embedded tissue sections were rehydrated in water, re-fixed in 4% formaldehyde, incubated in proteinase K solution (20 g/ml) for 5 min, washed two times in PBS, incubated for 10 min in an equilibration buffer and finally exposed for 60 min to a labeling buffer containing both FITC-labeled dUTP and terminal deoxynucleotidyl transferase. Control samples without the terminal deoxynucleotidyl transferase enzyme or treated by DNase were also prepared. TUNEL-labeled samples were then washed in saline–sodium citrate buffer and mounted in Vectashield with DAPI to visualize the nuclei. TUNEL-positive cells were counted in 20 cross-sections of seminiferous tubules under a fluorescent microscope. Ten tissue slides were analysed per testis and the numbers of TUNEL-positive cells were normalized per area and averaged as technical replicates. Twelve testes were analyzed in each group. In all the samples, the number of TUNEL positive cells was analysed by NIS Elements picture analyser.

### Histological analysis of the embryonic testis

The embryonic testes were dissected from 13.5 embryos and fixed in 4% formaldehyde in PBS. We prepared paraffin-embedded tissue section (5μM thick). The primordial germ cells were labelled by anti-SSEA1 antibody (ab16285) (Abcam,). In brief the tissue sections were deparaffinized, rehydrated and incubated in PBS for 5 min. The antigen retrieval was performed by heating the slides with sections in citrate buffer (IHC world general protocol) and HistoReveal (Abcam) according to the manufacturer protocol (5 min incubation at RT) and protein blocking was performed by Protein Block (Abcam) during 10 min at RT. The slides were subsequently incubated with the primary antibody (anti-SSEA1, Abcam, ab16285, 10 μg/ml) at 37°C for 60 min. After washing in PBS, slides were incubated with the secondary antibody (Goat Anti-Mouse IgM H&L, Abcam, 10 μg/ml) at 37°C for 60 min. After washing in PBS and water, the tissue slides were used for TUNEL assay according to the protocol described above with some modifications. In these samples no formalin fixation and proteinase K digestion were performed. Subsequently, slides were mounted with Vectashield (Vector) (DAPI counterstaining) and analysed under a fluorescent microscope. The numbers of TUNEL-positive and SSEA1-positive cells were counted with NIS Elements picture analyser and normalized to the sample area.

### RT-qPCR

MicroRNA expression was analysed by RT-qPCR from 10 ng of total RNA. We prepared complementary DNA by reverse transcription in a final volume of 15 μl containing 1X RT buffer, 0.25 mM of each dNTP, 50U of MultiScribeReverse Transcriptase (Applied Biosystems), 3U of RNase inhibitor and the specific stem-loop primer. The reaction was performed at 16°C for 30 min, 42°C for 30 min, 85°C for 5 min. The cDNAs resulting from the reverse transcription reaction were amplified by real-time quantitative PCR with the TaqMan universal PCR master mix using the following conditions: 10 min at 95°C followed by 40 cycles of 97°C for 30 s and 60°C for 1 min in the LightCycler 480 system (Roche).

mRNA expression was also performed by RT-qPCR. After heat-shock treatment at 95°C for 5 min, total RNA (100 ng) was retrotranscribed by adding 10 μl of a mix containing 2.5 μM Oligo dT17, 1X First-Strand Buffer (Invitrogen), 0.01 M dithiothreitol (DTT), 2U of RNase inhibitor (RNAsin, Promega), 0.5 mM of each dNTP and 200U of superscript II (Invitrogen). The reaction was completed to a final volume of 20 μl with RNase-free water. The cDNAs resulting from the reverse transcription reaction were amplified by real-time quantitative PCR. Reactions were performed by adding 7 μl of LightCycler 480 probes master mix (Roche) to each well containing 3 μl of template cDNA and 0.0625 μM of each specific primer in a 10 μl reaction volume. PCR profiles were obtained using the LightCycler 480 System (Roche) using the following PCR conditions: denaturation at 95°C for 10 min; 50 cycles of amplification of 15 s at 95°C, 30 s at 61.4° C and 1 min at 72°C. Synthetic oligonucleotides used for PCR were designed online with ProbeFinder version 2.20 (Roche) and purchased from Roche. Data was normalized using the 2-^ΔΔCt^ method [60] and *Sdha* and *Mapk1* to normalize the data [61]. We used the following primers for qPCR: Mapk1 ggattgaagttgaacaggctct and gaatggcgcttcagcaat; Sdha CAGTTCCACCCCACAGGTA and TCTCCACGACACCCTTCTGT; Tut4 (Zcchc11) CAGCAAAGAAAGCCACCAGT and AAAAGGCATTCCATCCATCA; Tut7(Zcchc6):CATTAAAAAGGAATGCCCACA and TTCTTTTTGTCTTCATGTAAAAGCAC; Blimp1 (Prmd1) TGCGGAGAGGCTCCACTA and TGGGTTGCTTTCCGTTTG; Lin28a ACATGCAGAAGCGAAGATCC and CCTTGGCATGATGGTCTAGC; Lin28bGAGTCCAGGATGATTCCAAGA and TGCTCTGACAGTAATGGCACTT; pri-let-7a CCCTGGATGTTCTCTTCA and TCACCTTAGGAAAGACAGTAGATT; mmu-let-7a-1-3p MIMAT0004620 TaqMan miRNA assay (Applied Biosystems); mmu-mir-21 MIMAT0004628: TaqMan miRNA assay (Applied Biosystems); mmu-mir-23bMIMAT0012538 TaqMan miRNA assay (Applied Biosystems).

### Methylation analysis

RRBS was performed on 100ng of genomic DNA prepared from PGCs or spermatozoa as described [62]. Final RRBS libraries were amplified with 17 cycles of PCR and subjected to paired-end sequencing (2x75bp) on an Illumina HiSeq2000 apparatus by Integragen SA. Sequencing reads were cleaned with Trim Galore and aligned to the mouse mm10 genome with BSMAP, allowing 4 mismatches. We only retained reads with a unique best hit in the genome. Methylation percent values were calculated with BSMAP as the ratio of the number of Cs over the total number of Cs and Ts. For all data analysis, we filtered CpGs to have a minimum sequencing depth of 8x. RRBS was performed on pools of PGCs purified from 30 testes. For sperm, we performed RRBS in triplicates on sperm pooled from three exposed animals, as well as five control pools from unexposed animals. To find differences in DNA methylation, we used the methylkit R package to search for changes in methylation greater than 20% in 400bp tiles containing at least 3 CpGs. All data processing and representation were performed with the R software (http://www.r-project.org). The RRBS data is available at the NCBI Gene Expression Omnibus database under accession number GSE65784 For gene-specific analysis of DNA methylation, we performed bisulfite conversion of genomic DNA with the Epitect kit (Qiagen). Subsequently, PCR amplifications were performed at regions of interest followed by cloning, as described [63]. We used the following primers for bisulfite PCR: Lin28a GGGTTTTTTGTATTTTAAGGTGTGTT and AAAATCCCCATCTCCAAATATATATATATAA; Lin28b TTTTATGAGATTAGAGAAGTTTGTGTGTAG and TCCAAAAATAAAAACTTTCCCTTTAAAC.

## Supporting Information

S1 Fig Cartoon representation of the experimental model approach of VCZ exposure used and the cells, tissues and parameters assessed in each generation. (TIF)

S2 Fig Expression of terminal uridylyltransferases (TUTases) in germ cell precursors of mice exposed to VCZ. The graphs show the expression of *Tut4* (*A*) and *Tut7* (*B*) in 13.5 dpc PGCs from VD1 and VD2 exposed embryos relative to the unexposed control (log2 of fold change of expression) along the three generations. (a) indicates a significant statistical difference of VD1 and VD2 compared to control (p≤0.01); (b) indicates a significant statistical difference of VD1 compared to VD2 (p≤0.01). The error bars represent the standard deviation (SD).
(TIF)

S3 Fig Methylation profiles in sperm from control F1 males and F1 males exposed to the high dose of Vinclozolin (VCZ). The graphs show RRBS methylation scores at single CpGs in the exon 18 of the *Taok3* gene (*A*) and at known maternal germline DMRs (gDMRs) of imprinted loci (*B*). In B, the green bars depict the position of the gDMR.
(TIFF)

S1 Table DNA methylation sequencing statistics. (DOC)
