# Supplementary figures and images for: Exposure to Endocrine Disruptor Induces Transgenerational Epigenetic Deregulation of MicroRNAs in Primordial Germ Cells
Source: PLoS One. 2015 Apr 21;10(4):e0124296. doi: 10.1371/journal.pone.0124296 (PMC4405367; doi:10.1371/journal.pone.0124296)

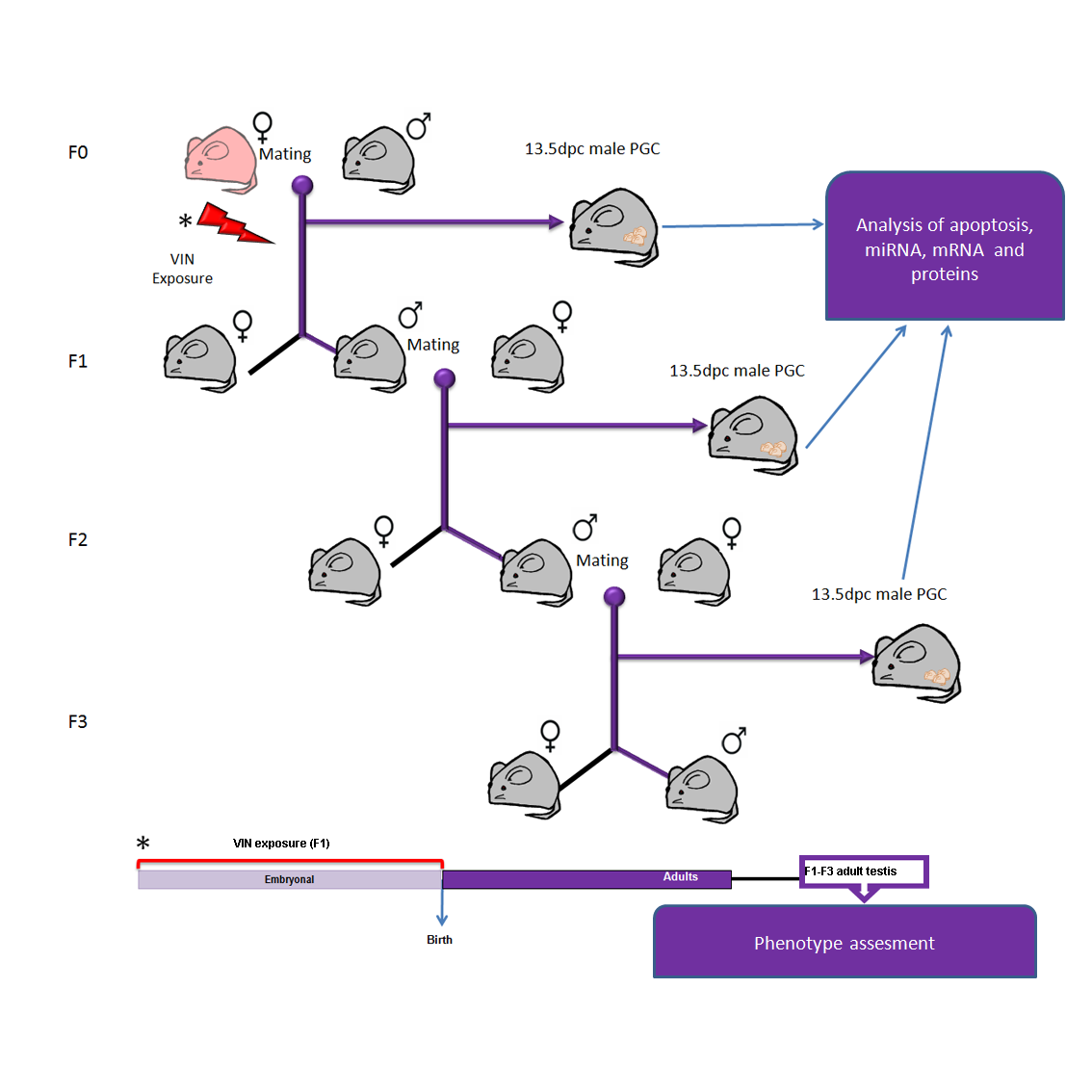

Supplement: S1 Fig — (TIF) [file pone.0124296.s001.tif]

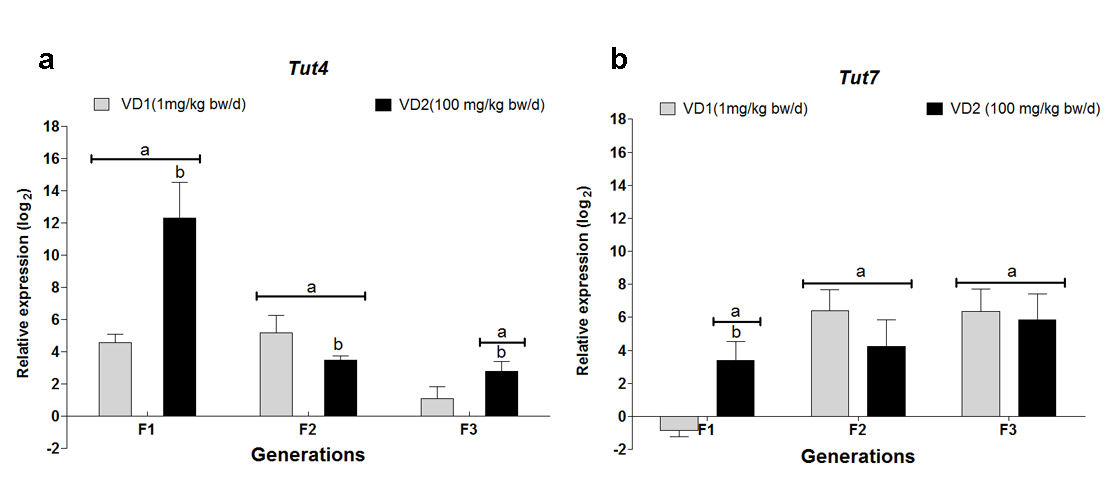

Supplement: S2 Fig — The graphs show the expression of Tut4 (A) and Tut7 (B) in 13.5 dpc PGCs from VD1 and VD2 exposed embryos relative to the unexposed control (log2 of fold change of expression) along the three generations. (a) indicates a significant statistical difference of VD1 and VD2 compared to control (p≤0.01); (b) indicates a significant statistical difference of VD1 compared to VD2 (p≤0.01). The error bars represent the standard deviation (SD). (TIF) [file pone.0124296.s002.tif]

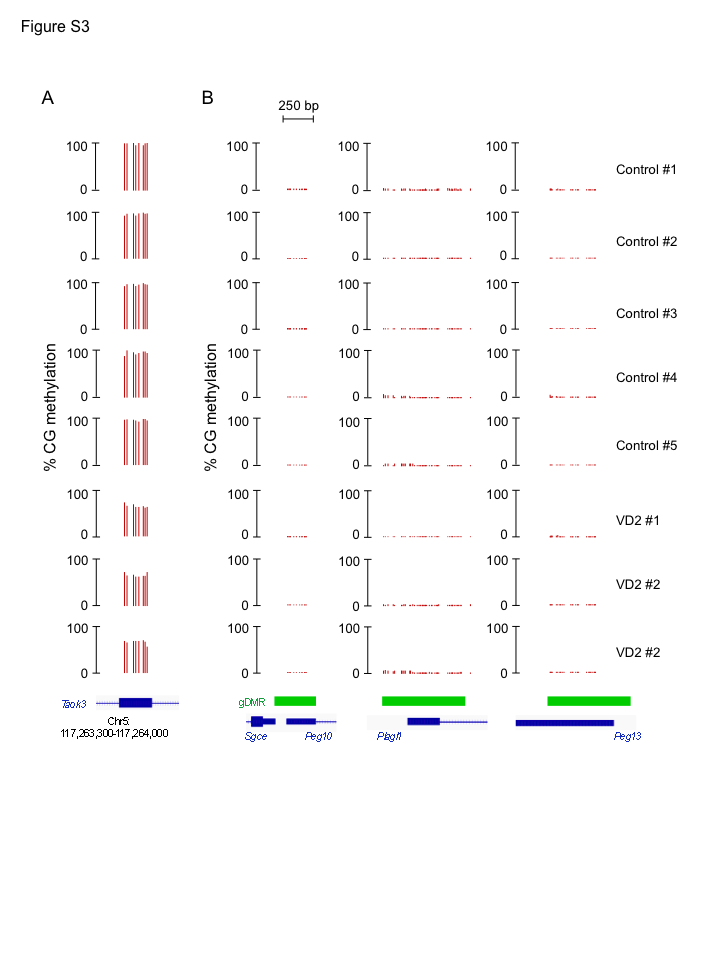

Supplement: S3 Fig — The graphs show RRBS methylation scores at single CpGs in the exon 18 of the Taok3 gene (A) and at known maternal germline DMRs (gDMRs) of imprinted loci (B). In B, the green bars depict the position of the gDMR. (TIFF) [file pone.0124296.s003.tiff]
