# Supplementary material for: Exposure to Endocrine Disruptor Induces Transgenerational Epigenetic Deregulation of MicroRNAs in Primordial Germ Cells
Source: PLoS One. 2015 Apr 21;10(4):e0124296. doi: 10.1371/journal.pone.0124296 (PMC4405367; doi:10.1371/journal.pone.0124296)
Supplement: S1 Table — (DOC) [file pone.0124296.s004.doc]

| **Sample** | **Paired-end reads** | **Mapping efficiency (%)** | **Reads with unique hits (%)** | **Estimated conversion rate (%)** | **CpGs 1x** | **CpGs 8x** | **Median sequencing depth** |
| --- | --- | --- | --- | --- | --- | --- | --- |
| **PGCs control#1** | 29,602,025 | 89.43 | 69.34 | 99.91 | 1,519,279 | 1,273,335 | 63x |
| **PGCs control#2** | 26,459,394 | 89.31 | 69.88 | 99.88 | 1,501,617 | 1,256,928 | 58x |
| **PGCs VD1** | 29,483,225 | 89.62 | 69.71 | 99.88 | 1,494,118 | 1,263,741 | 64x |
| **PGCs VD2** | 25,952,231 | 90.16 | 70.74 | 99.90 | 1,447,293 | 1,220,629 | 55x |
| **Sperm control#1** | 41,614,839 | 92.51 | 71.29 | 99.67 | 1,506,335 | 1,285,697 | 80x |
| **Sperm control#2** | 27,370,903 | 92.39 | 71.59 | 99.78 | 1,474,147 | 1,232,252 | 58x |
| **Sperm control#3** | 23,916,584 | 92.71 | 70.31 | 99.79 | 1,477,737 | 1,241,280 | 49x |
| **Sperm control#4** | 24,186,087 | 92.14 | 72.44 | 99.80 | 1,532,820 | 1,234,934 | 46x |
| **Sperm control#5** | 49,550,935 | 91.76 | 71.56 | 99.65 | 1,611,355 | 1,355,501 | 98x |
| **Sperm VD2#1** | 26,074,125 | 92.11 | 72.79 | 99.77 | 1,523,119 | 1,295,320 | 56x |
| **Sperm VD2#2** | 29,206,596 | 89.26 | 70.81 | 99.76 | 1,545,102 | 1,260,722 | 53x |
| **Sperm VD2#3** | 29,027,138 | 90.97 | 72.16 | 99.88 | 1,578,010 | 1,306,076 | 56x |

**Table S1. DNA methylation sequencing statistics**
